# Terpyridine platinum compounds induce telomere dysfunction and chromosome instability in cancer cells

**DOI:** 10.18632/oncotarget.28020

**Published:** 2021-07-20

**Authors:** Nikolai Petrov, Hee-Sheung Lee, Mikhail Liskovykh, Marie-Paule Teulade-Fichou, Hiroshi Masumoto, William C. Earnshaw, Yves Pommier, Vladimir Larionov, Natalay Kouprina

**Affiliations:** ^1^Developmental Therapeutics Branch, National Cancer Institute, National Institutes of Health, Bethesda, MD 20892, USA; ^2^Chemistry and Modelling for the Biology of Cancer, CNRS UMR 9187-INSERM U1196 Institute Curie, Research Center, Campus University Paris-Saclay, Orsay, France; ^3^Laboratory of Chromosome Engineering, Department of Frontier Research and Development, Kazusa DNA Research Institute, Kisarazu, Chiba 292-0818, Japan; ^4^Wellcome Centre for Cell Biology, School of Biological Sciences, King's Buildings, University of Edinburgh, Max Born Crescent, Edinburgh EH9 3BF, Scotland

**Keywords:** chromosome instability, CIN, platinum-derived G4-quadruplexes, telomere dysfunction, human artificial chromosome

## Abstract

Telomerase/telomere-targeting therapy is a potentially promising approach for cancer treatment because even transient telomere dysfunction can induce chromosomal instability (CIN) and may be a barrier to tumor growth. We recently developed a dual-HAC (Human Artificial Chromosome) assay that enables identification and ranking of compounds that induce CIN as a result of telomere dysfunction. This assay is based on the use of two isogenic HT1080 cell lines, one carrying a linear HAC (containing telomeres) and the other carrying a circular HAC (lacking telomeres). Disruption of telomeres in response to drug treatment results in specific destabilization of the linear HAC. Results: In this study, we used the dual-HAC assay for the analysis of the platinum-derived G4 ligand Pt-tpy and five of its derivatives: Pt-cpym, Pt-vpym, Pt-ttpy, Pt(PA)-tpy, and Pt-BisQ. Our analysis revealed four compounds, Pt-tpy, Pt-ttpy, Pt-vpym and Pt-cpym, that induce a specific loss of a linear but not a circular HAC. Increased CIN after treatment by these compounds correlates with the induction of double-stranded breaks (DSBs) predominantly localized at telomeres and reflecting telomere-associated DNA damage. Analysis of the mitotic phenotypes induced by these drugs revealed an elevated rate of chromatin bridges (CBs) in late mitosis and cytokinesis. These terpyridine platinum-derived G4 ligands are promising compounds for cancer treatment.

## INTRODUCTION

Normal human somatic cells contain 46 chromosomes. A distinguishing feature of many cancer cells is whole-chromosomal instability (CIN) manifested as unequal chromosome distribution during cell division. As a consequence, the number of chromosomes deviates from the modal number of 46. Such chromosome mis-segregation can lead to large-scale changes in gene copy number and gene expression levels.

Analysis of the consequences of induced increases in chromosome segregation errors on the viability of tumor cells led to the breakthrough discovery that chromosome mis-segregation may be exploited therapeutically [1]. That study revealed that there is a threshold level beyond which CIN becomes a barrier to tumor growth. Moreover, the authors reported that treatment with drugs that target the mitotic checkpoint or interfere with chromosome alignment enhances the amount and severity of chromosome segregation errors and leads to selective killing of tumor cells. Subsequent studies confirmed that interference with chromosome segregation can push genetically unstable cancer cells towards death, whereas more stable non-transformed cells are better able to tolerate such insults [2–4].

Telomerase/telomere-targeting therapy is considered to be a potentially promising approach for cancer treatment [5–8] because even transient telomere dysfunction can induce chromosomal instability in human cells [9]. Telomeres are protein-DNA complexes located at the ends of eukaryotic chromosomes. They protect the DNA ends against exonucleases and ligases and prevent inappropriate chromosome end-to-end fusion [10]. Telomere length maintenance is required for long-term “healthy” division of cells. The enzyme telomerase elongates telomeres and maintains a telomere length equilibrium that prevents telomeres from becoming critically short [11]. Because telomeres contain G-rich repetitive sequences, they can form four-stranded structures called G-quadruplexes (G4s) [12–14]. A variety of data argues that such structures may have important effects on genomic stability [14, 15]. In particular, formation of G4s at telomeres could impede telomerase recognition and inhibit telomere elongation leading to telomere shortening [16, 17]. Excessively short telomeres no longer protect chromosome ends and cells undergo senescence or apoptosis [18–22]. However, till now only a limited number of chemical compounds that target telomerase or telomeres have been identified and only a few are in clinical trials. Thus, telomeres are promising targets for discovery of ligands that stabilize G4s at telomeres, thereby perturbing telomere maintenance and leading to genomic instability [23–27]. Such chemical compounds targeting G4 structures at telomeres could be potentially useful for anticancer therapy [7, 28–31]. Known G4 ligands include metal complexes that are promising compounds employed for cancer treatment [32–35].

**Figure 1:**
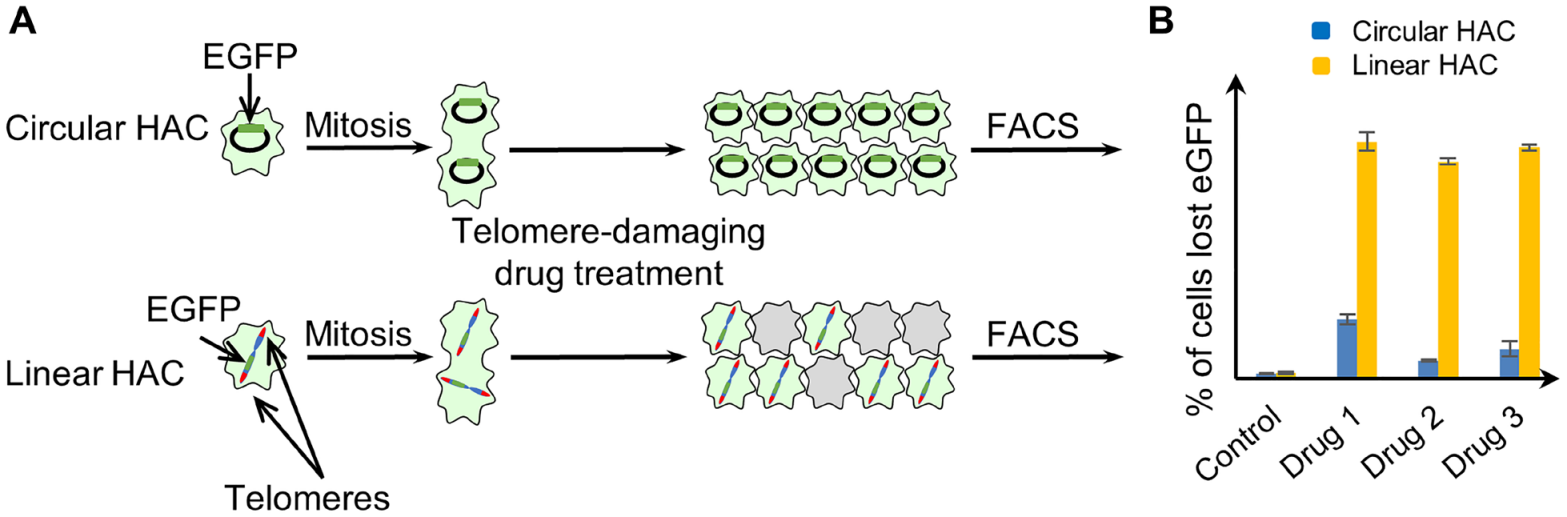
Experimental design for measuring CIN induced by telomere disfunction, based on the use of a linear EGFP-HAC versus a circular EGFP-HAC. (**A**) Human fibrosarcoma HT1080 cells carried either the circular alphoid^tetO^-HAC [38] or the linear 21DqHAC [37]. Cells that inherit a HAC display green fluorescence, while cells that lack a HAC do not. Both HACs are stable during cell division. Therefore, the control cells without drug treatment display uniform green fluorescence while in the drug-treated population there is a mixture of EGFP positive and negative cells. (**B**) The actual percentage of cells carrying an EGFP-HAC was measured by FACS as previously described [36].

Recently we developed a dual-HAC-based assay allowing quantitative comparison of the efficiency and specificity of compounds to induce telomere dysfunction [36]. This assay is based on the use of two cell lines, one of which carries a linear EGFP-expressing HAC (containing telomeres) [37]. The other carries a circular EGFP-expressing HAC (lacking telomeres) [38] (Supplementary Figure 1). Compounds targeting telomeres should lead to selective destabilization of the linear HAC and loss of the EGFP signal (Figure 1). In contrast, compounds that destabilize both the linear and circular HACs are likely to have off-target effects in addition to any effect on telomeres (for example on kinetochores or the mitotic spindle). This dual-HAC assay was previously applied to analyze a set of compounds, including 19 G-quadruplex ligands, and identify those that specifically interfere with telomeres [36]. As a result, the compounds were ranked according to their potency at destabilizing the linear HAC. The assay revealed that two G4 stabilizing metal complexes, Cu-ttpy and Pt-ttpy, exhibited the highest rate of linear HAC mis-segregation [36]. We demonstrated an increased number of double-strand DNA breaks associated with telomeres after Cu-ttpy and Pt-ttpy treatment. This telomere damage led to the formation of chromosome bridges that ultimately resulted in chromosome mis-segregation.

In this study, we used the dual-HAC assay to analyze a larger set of platinum complexes comprising either a simple terpyridine moiety (Pt-tpy) or various terpyridine moieties with extended surface areas, i.e., Pt-ttpy, Pt-BisQ, Pt-vpym, Pt-cpym, and Pt(PA)-tpy [39]. It was shown previously that expanding the surface of the terpyridine core in this series of platinum complexes can both increase their affinity for telomeric G4s [40] and modulate their capacity to induce specific metalation of G4s *in vitro* [39]. However, it was not known how these structurally modified Pt-tpy derivatives would affect genomic stability. We have therefore explored the genomic activity of this expanded set of compounds in a more biologically relevant setting (the dual-HAC assay) and compared them to the previously studied Pt-ttpy derivative [36]. We found that treatment of cancer cells with either Pt-cpym, Pt-vpym, Pt-ttpy or Pt-tpy induces telomere dysfunction leading to high levels of chromosome instability. Thus, these terpyridine platinum compounds potentially serve as onco-therapeutic agents for cancers.

## RESULTS

### A dual-HAC assay revealed new platinum-derived G4 ligands that affect a linear HAC stability

**Figure 2:**
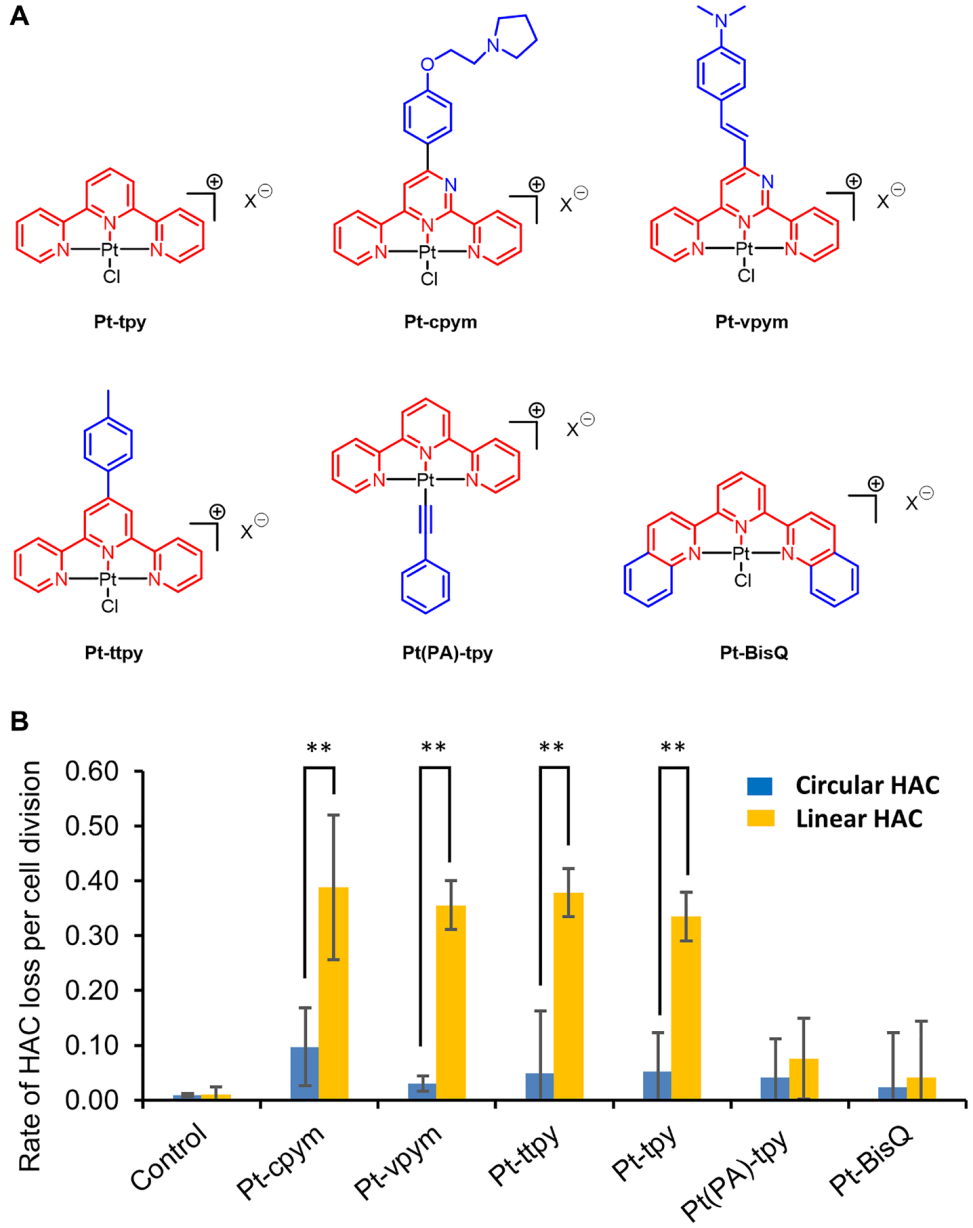
Effect of terpyridine platinum compounds on a linear versus a circular EGFP-HAC mis-segregation rate. (**A**) The chemical structures of Pt-tpy and its five derivatives Pt-cpym, Pt-vpym, Pt-ttpy, Pt(PA)-tpy, and Pt-BisQ; the terpyridine (tpy) core is shown in red and the aromatic surface expansion performed for each derivative is shown in blue. (**B**) HT1080 cells containing either a linear or a circular HAC were treated by Pt-tpy or its derivatives at concentrations corresponding to their LC_50_ (Supplementary Table 1) overnight. The rate of HAC loss was calculated based on the ratio of HAC-positive to HAC-negative cells as described in Materials and Methods. The controls correspond to the frequency of spontaneous loss of the linear or circular HAC in HT1080 cells. The highest rate of the linear HAC loss was observed after treatment by Pt-cpym, Pt-vpym, Pt-ttpy, and Pt-tpy. ^*^Indicates *P*-value < 0.05; ^**^ *P* < 0.01.

In this work, we analyzed the platinum-derived G-quadruplex Pt-tpy ligand and five recently developed derivatives, i.e., Pt-cpym, Pt-vpym, Pt-ttpy, Pt(PA)-tpy, and Pt-BisQ [39]. Chemical structures of these terpyridine platinum compounds are shown in Figure 2A. We used the dual-HAC-based assay to determine how the various structural modifications alter the effects of the different Pt-tpy derivatives on chromosome stability.

As previously demonstrated in other screens, the highest rate of HAC loss occurs at the compound’s LC_50_ [41]. Thus, the LC_50_ provides a parameter to normalize the results from different drugs/compounds. Therefore, first we determined LC_50_ values for Pt-tpy, Pt-cpym, Pt-vpym, Pt-ttpy, Pt(PA)-tpy, and Pt-BisQ. The results are shown in Supplementary Table 1. Figure 2B illustrates the effect of these compounds on the rate of loss of circular versus linear HACs. As seen, no significant increase in loss of either HAC was detected for Pt(PA)-tpy and Pt-BisQ. Worth noting is that the rather high cytoxicity of Pt(PA)-tpy limited its proper evaluation in cells. Four other compounds, Pt-cpym, Pt-vpym, Pt-ttpy and Pt-tpy exhibited strong effects on stability of the linear but not the circular HAC, comparable to that described earlier for Pt-ttpy [36]. After treatment with those compounds, the rate of the linear HAC loss was almost 4-fold (Pt-cpym), 11.8-fold (Pt-vpym), 7.7-fold (Pt-ttpy), and 6.4-fold (Pt-tpy) higher than that observed in control cells containing the circular HAC (Figure 2B and Supplementary Table 2). Notably, four compounds have a similar square planar geometry due to platinum (II) tris-coordination to the terpyridine moiety (Figure 2A). This is in agreement with our previous observation that the planar shape resulting from the metal center geometry strongly influences the ability of metal-terpyridine complexes to discriminate quadruplex from duplex-DNA [40].

Thus, the dual-HAC assay allowed us to identify new compounds that potentially target telomeres and, therefore, are promising candidates for future development as therapeutic agents. We decided to investigate further the mechanism of action of Pt-cpym, Pt-vpym, Pt-ttpy, and Pt-tpy, as these exhibited the largest effects on linear HAC stability in cancer cells.

### Treatment by Pt-cpym, Pt-vpym, Pt-ttpy, and Pt-tpy leads to micronucleus formation

Micronuclei (MNi) are formed when chromosome fragments or whole chromosomes lag behind at anaphase during cell division and are not incorporated into the nucleus with the bulk of the segregated chromatids. MNi typically occur as a consequence of mutations or drug treatments that interfere with chromosome segregation. Thus, MNi scoring is a simple and rapid method to assess chromosome instability [42, 43]. We, therefore, used a micronucleus formation assay to evaluate if Pt-tpy and its derivatives Pt-cpym, Pt-vpym, and Pt-ttp affect the stability of natural chromosomes in human fibrosarcoma HT1080 cells. In our assay, we inhibited cytokinesis using cytochalasin B. This allowed us to conclude that binucleated cells had gone through mitosis during the period of drug treatment (see Materials and Methods for detail). Only binucleated cells were analyzed for the presence of MNi.

**Figure 3:**
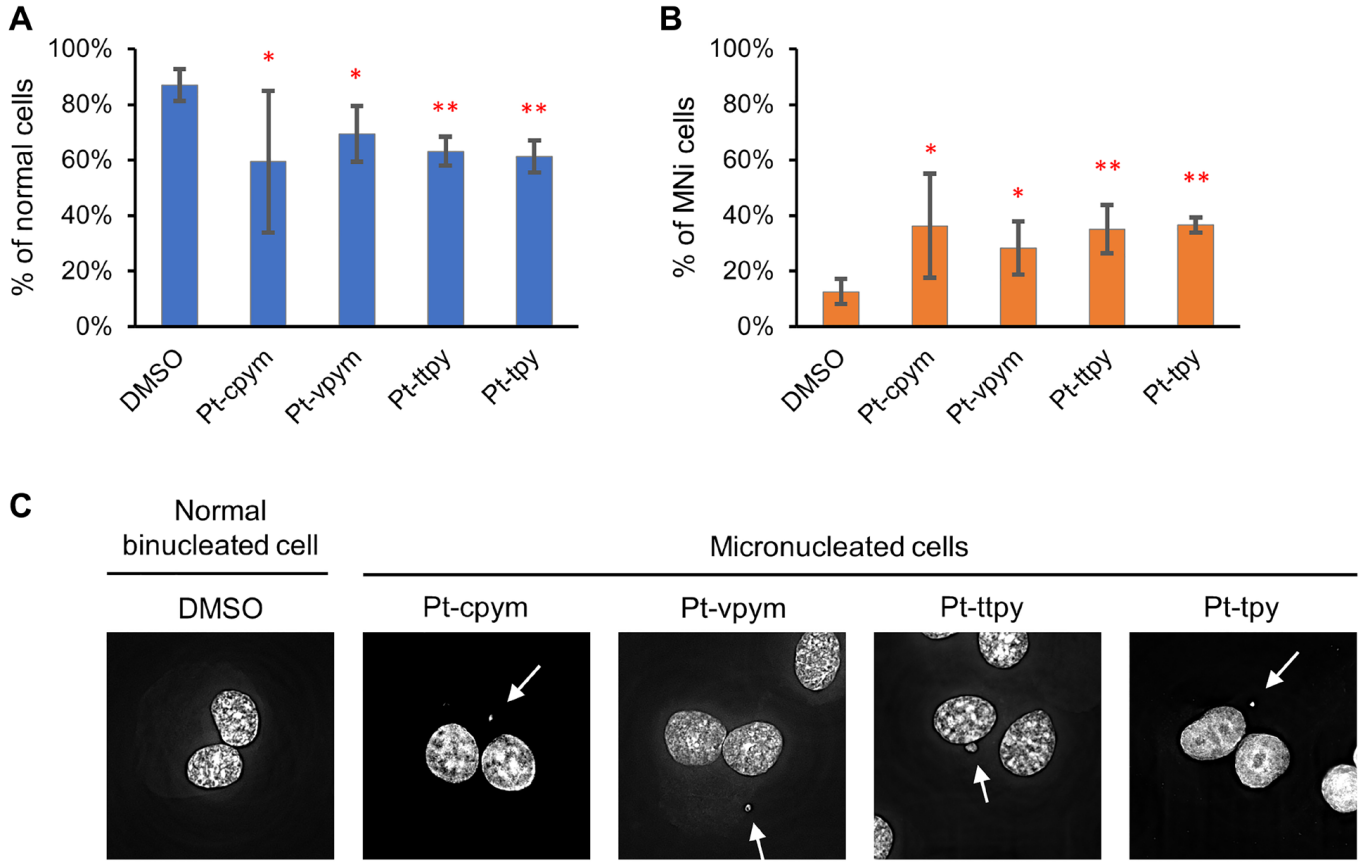
Micronucleus formation in HT1080 cells after treatment by Pt-tpy and its three derivatives, Pt-cpym, Pt-vpym and Pt-ttpy. (**A**) The percentage of binucleated cells without abnormalities. (**B**) The percentage of cells with MNi. Error bars correspond to the 95% confidence intervals (CI95%) of four replicates. Red asterisks indicate statistical significance when compared to the control [calculated using the two-tailed Student’s *t*-test with Bonferroni correction for multiple testing (^*^ *P* < 0.05; ^**^ *P* < 0.01)]. (**C**) Representative images of a normal binucleated cell; binucleated cells containing MNi. White arrows point to MNi. The cells were stained with DAPI and Eosin Y.

Concentrations of the compounds used in these experiments are presented in Supplementary Table 1. Our analysis revealed a significant difference in MNi formation between cells treated with the compounds versus untreated (DMSO-treated negative control cells) (Figure 3A, 3B and 3C and Supplementary Table 3). Data in Figure 3B are expressed in terms of percentages of the cells with MNi observed on the total number of binucleated cells analyzed and in terms of fold induction comparing treated versus untreated samples (DMSO). The percentage of MNi formation for Pt-cpym was 36% (95% CI 18–55%; 2.8-fold increase compared to the negative control), for Pt-vpym 28% (95% CI 19–38%, 2.2-fold increase), for Pt-ttpy 35% (95% CI 26–44%; 2.8-fold increase) and for Pt-tpy 37% (95% CI 34–39%; 2.9-fold increase), correspondingly.

To summarize, MNi analysis confirmed that Pt-tpy and its derivatives have significant effects on the accuracy of mitotic transmission of the natural chromosomes. The elevated frequencies of binucleated cells with MNi support our assumption that these compounds have a potential to be used as therapeutic agents.

### Treatment by Pt-cpym, Pt-vpym, Pt-ttpy, and Pt-tpy induces chromatin bridges in late mitosis

Formation of chromatin bridges is a sensitive measure of chromosome damage leading to chromosomal instability [44]. Chromatin bridges can form when telomeres of sister chromatids fuse and fail to completely segregate into the respective daughter cells [10]. Therefore, firstly, we scored the formation of CBs stained with DAPI after mitosis. This is a relatively insensitive method that identifies only larger chromatin bridges. In these experiments, we observed no statistically significant increase in the percentage of cells showing CBs in cells treated with any of the drugs (Supplementary Figure 2 and Supplementary Table 3).

**Figure 4:**
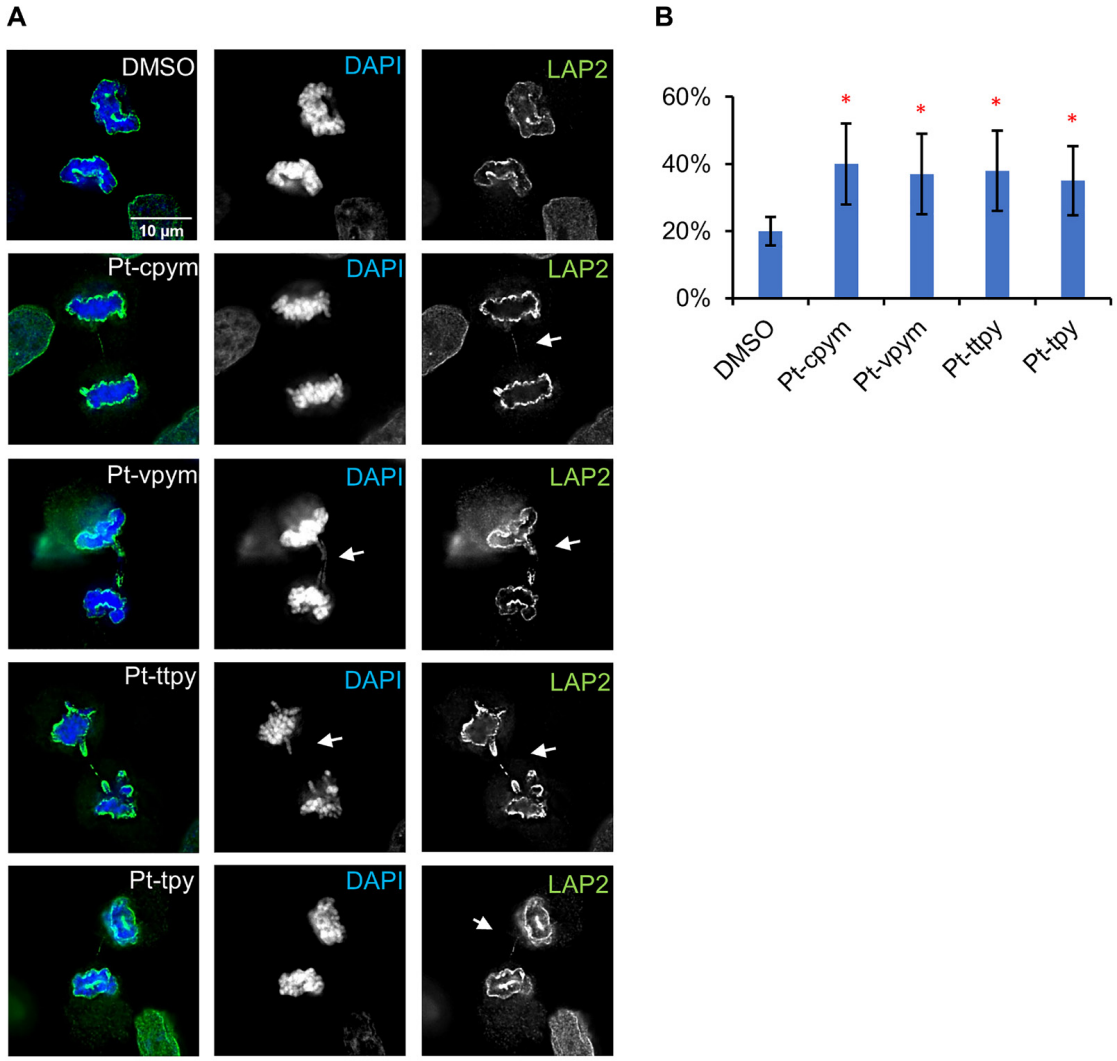
Chromatin bridge formation in HT1080 cells after treatment by Pt-tpy and its three derivatives, Pt-cpym, Pt-vpym and Pt-ttpy at telophase/cytokinesis. (**A**) Cells after drug treatment were stained with antibodies against LAP2. LAP2 allows visualization and quantification of fine chromatin bridges that are too thin to be spotted with DAPI staining. (**B**) The percentage of cells presenting chromatin bridges in telophase/cytokinesis upon treatment with Pt-cpym, Pt-vpym, Pt-ttpy, and Pt-tpy quantified using LAP2 as a marker. ^*^Indicates *P*-value < 0.05; ^**^ *P* < 0.01.

To further analyze if thinner CBs appear in telophase, we used antibodies against LAP2, a nuclear envelope protein that decorates the chromatin (including chromatin bridges) from late anaphase onwards while it is still in the process of moving polewards. Because LAP2 coats the surface of any chromatin bridges during anaphase, it can render visible even bridges whose DNA content is too low to be seen by conventional DAPI staining. This experiment thus allows a more precise quantification of the frequency of CBs after drug treatment. Indeed, with LAP2 antibody, CBs were readily visible, even when they were too fine to be visualized by DAPI staining (Figure 4A).

This analysis revealed a statistically significant difference in the frequency of CBs observed in drug-treated cells versus untreated (DMSO) (Figure 4B and Supplementary Table 4). The percentage of CBs formation for Pt-cpym was 40% (95% CI 16–24%; 2.1-fold increase compared to the negative control), for Pt-vpym 37% (95% CI 25–49%, 1.9-fold increase), for Pt-ttpy 38% (95% CI 26–50%; 1.9-fold increase) and for Pt-tpy 35% (95% CI 25–45%; 1.8-fold increase). This increase in chromatin bridges is consistent with disruption of telomere function.

### Treatment by Pt-cpym, Pt-vpym, Pt-ttpy, and Pt-tpy leads to an increased number of double-stranded breaks that colocalize with telomeric markers

To investigate further the mechanism(s) by which treatment with these drugs leads to chromosome instability, we stained HT1080 cells with an antibody against phosphorylated histone γH2AX to detect formation of DNA double-stranded breaks (DSBs).

**Figure 5:**
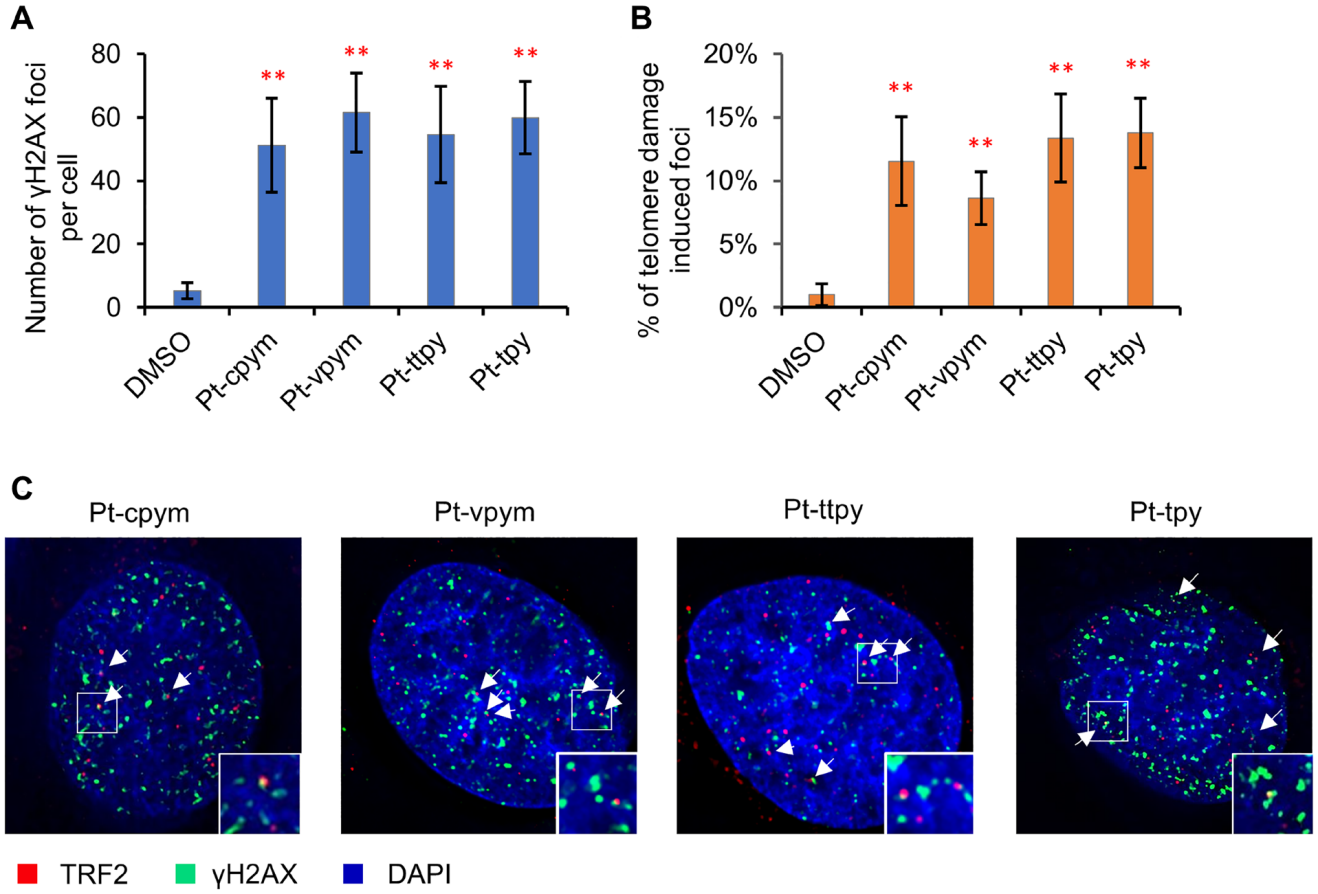
Immunostaining of DNA double stranded breaks with an antibody against γH2AX and the telomeric protein TRF2. (**A**) The percentage of γH2AX foci in cells treated with Pt-tpy and its three derivatives, Pt-cpym, Pt-vpym and Pt-ttpy. Accumulation of γH2AX foci occurred at day 3 in all cases. (**B**) Co-localization of γH2AX foci with the telomeric protein TRF2. This panel shows the percentage of γH2AX foci present in the telomeric sequences. (**C**) Examples of immunostaining of cells treated with the compounds. Green signals – γH2AX staining as a marker for DSBs. White arrows point to cell nuclei with γH2AX signals. Red signals – TRF2 as a marker for telomere localization. A statistically significant co-localization of γH2AX foci and the TRF2 protein was observed at day 3 (*t*-test with Bonferroni correction: ^*^ *P* < 0.05; ^**^ *P* < 0.01) when compared to a negative control (DMSO) (Supplementary Table 6).

Concentrations of the compounds used in this experiment are presented in Supplementary Table 1. The number of DSBs were expressed in terms of the number of the γH2AX foci per nucleus and in terms of fold induction comparing treated versus untreated samples (DMSO). A statistically significant increase in the number of γH2AX foci in interphase was observed in cells after treatment with all four compounds (Figure 5A and Supplementary Table 5). The effect observed was as follows: Pt-cpym, 51.1 foci/cell (95% CI 36.3–65.9 foci/cell; 9.6-fold increase) compared to the control level of DNA damage in HT1080 cells which was 5.3 foci/cell (95% CI 2.8–7.9 foci/cell). After Pt-vpym treatment, there were 61.5 foci/cell (95% CI 49–74 foci/cell; 11.6-fold increase). After Pt-ttpy treatment there were 54.5 foci/cell (95% CI 39.3–69.7 foci/cell; 10.3-fold increase). After Pt-tpy treatment, there were 59.9 foci/cell (95% CI 48.5–71.2 foci/cell; 11.3-fold increase). Thus, in HT1080 cells chromosome instability after treatment by Pt-cpym, Pt-vpym, Pt-ttpy, and Pt-tpy is accompanied by induction of DSBs.

We next examined whether the γH2AX foci co-localize with or are adjacent to the telomeric protein TRF2 (telomeric repeat binding factor 2) in order to assess whether the DNA damage is associated with telomeric sequences. Previously we demonstrated that Pt-ttpy was incorporated into telomeres *in vivo* and caused DNA breaks [36]. Therefore, Pt-ttpy was used as an internal positive control in these experiments. Immunostaining with antibodies against TRF2 and γH2AX was carried out at day 3 (72 hrs) of drug treatment (see Materials and Methods). The level of telomere-specific DNA damage is expressed in terms of the frequency of the γH2AX foci that colocalize with telomere-specific TRF2 signals and in terms of fold induction comparing treated versus untreated samples (DMSO).

We observed that a 72 hrs treatment with any of the four compounds induced a statistically significant increase in co-localization of γH2AX foci with TRF2. The percentage of TRF2/γH2AX-positive foci in HT1080 increased from 1.0% (95% CI 0.2–1.8%) in DMSO-treated negative control cells (spontaneous DNA damage) up to 11.5% (95% CI 8.1–15%; 11.5-fold increase) in Pt-cpym-treated cells, 8.6% (95% CI 6.6–10.7%; 8.6-fold increase) in Pt-vpym-treated cells, 13.4% (95% CI 9.9–16.8%; 13.4-fold increase) in Pt-ttpy-treated cells and 13.8% (95% CI 11.3–16.5%; 13.8-fold increase) in Pt-tpy-treated cells (Figure 5B and 5C and Supplementary Table 6). Since telomeric DNA only represents about 1/6,000^th^ of the total genomic DNA, this increase above background is likely to be highly significant.

These data suggest that chromosome loss in Pt-cpym, Pt-vpym, Pt-ttpy, and Pt-tpy treated cells may be a consequence of the induction of DSBs predominantly localized at or near telomeres.

### Treatment by Pt-cpym, Pt-vpym, Pt-ttpy, and Pt-tpy induces the formation of telomere aberrations

Recombination events, which resolve replication fork collapse, can generate telomere aberrations such as telomere doublets (also named Multiple Telomeric Signals or MTSs), telomere fusions (also named Sister Chromatid Telomeric Fusions or SCTF), telomere loss and deletions. It has been reported that telomere doublets are linked to replicative defects of telomeres [45].

We analyzed HT1080 cells after treatment by Pt-cpym, Pt-vpym, Pt-ttpy, and Pt-tpy to ask whether treatment with these compounds was associated with increases in this class of chromosomal aberrations. For this purpose, metaphase spreads after 3 days of treatment were prepared and then labeled with a telomere specific PNA probe (see Materials and Methods for details) and counterstained with DAPI. Concentrations of the compounds used in these experiments are presented in Supplementary Table 1. Data are expressed in terms of frequencies of aberrations per metaphase analyzed and in terms of fold induction comparing treated versus untreated samples (DMSO).

**Figure 6:**
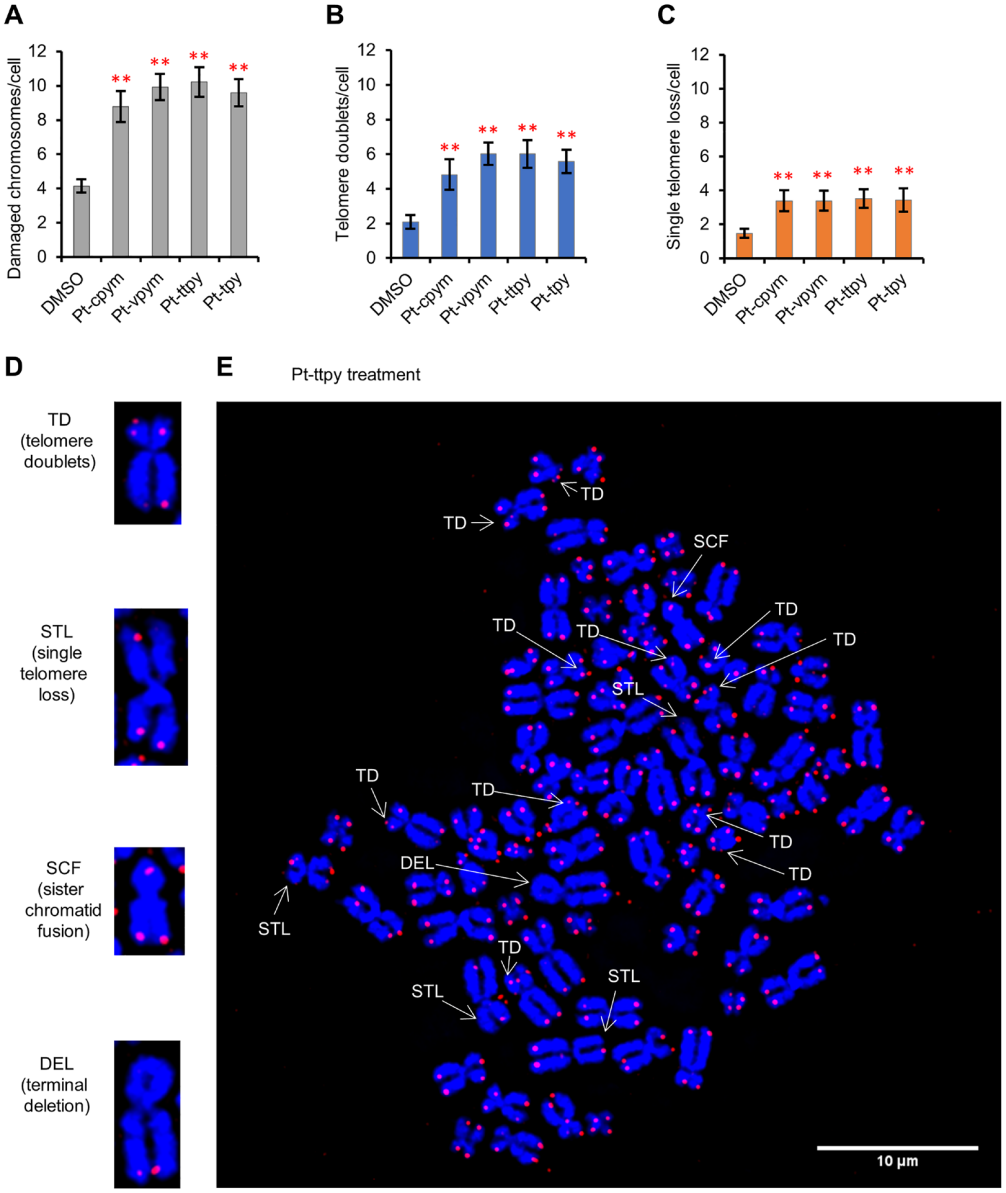
Specific telomere aberrations in HT1080 cells induced by treatment of Pt-tpy and its derivatives, Pt-cpym, Pt-vpym and Pt-ttpy. (**A**–**C**) Histograms show the percentages of chromosomes with the indicated telomere damage per cell detected in metaphase spreads of treated versus untreated cells (DMSO) hybridized with a telomeric PNA probe (in red) and then counterstained with DAPI (in blue). ^*^Indicates a *t*-test *P*-value < 0.05; ^**^ *P* < 0.01. (**D** and **E**) Representative images of the different telomere aberrations after Pt-ttpy treatment. TD-telomere doublets; STL-single telomere loss; SCF-sister chromatid fusion; DEL-terminal deletion.

FISH with the telomeric probe on metaphase spreads showed that Pt-cpym, Pt-vpym, Pt-ttpy, and Pt-tpy treatment induces a broad spectrum of telomeric aberrations (Figure 6A, 6D, 6E and Supplementary Figure 3). Total number of aberrations increased to 8.8 per cell (95% CI 7.9–9.7 /cell; 2.1-fold increase) in Pt-cpym-treated cells versus untreated cells (4.1 aberrations/cell; 95% CI 3.8–4.5 /cell). After Pt-vpym treatment the number of aberrations was 9.9 per cell (95% CI 9.2–10.7 /cell; 2.4-fold increase), after Pt-ttpy treatment 10.2 aberrations per cell (95% CI 9.4–11.1 /cell; 2.5-fold increase), and after Pt-tpy treatment 9.6 aberrations per cell (95% CI 8.8–10.4 /cell; 2.3-fold increase) (Figure 6A and Supplementary Table 7).

More specifically, the number of chromosomes with telomere doublets (TD) increased significantly after treatment with each of the four drugs studied (Figure 6B). While in the control DMSO-treated cells we observed 2.1 TD per cell (95% CI 1.7–2.5 TD/cell), this number increased to 4.8 TD/cell (95% CI 3.9–5.7 TD/cell; 2.3-fold increase comparing to DMSO-treated cells) in Pt-cpym-treated cells, 6 TD/cell (95% CI 5.4–6.7 TD/cell; 2.9-fold increase) in Pt-vpym-treated cells, 6 TD/cell (95% CI 5.2–6.8 TD/cell; 2.9-fold increase) in Pt-ttpy-treated cells and 5.6 TD/cell (95% CI 4.9–6.3 TD/cell; 2.7-fold increase) in Pt-tpy-treated cells (Figure 6B and Supplementary Table 7).

Single telomere loss (STL) increased significantly after Pt-cpym treatment to 3.4 STL/cell (95% CI 2.8–4 STL/cell, 2.3-fold increase), after Pt-vpym treatment to 3.4 STL/cell (95% CI 2.8–4 STL/cell; 2.3-fold increase), after Pt-ttpy to 3.5 STL/cell (95% CI 3–4.1 STL/cell, 2.4–fold increase), and after Pt-tpy treatment to 3.4 STL/cell (95% CI 2.7–4.1 STL/cell; 2.3-fold increase) relative to control cells (1.5 STL/cell; 95% CI 1.2–1.7 STL/cell) (Figure 6C and Supplementary Table 7).

Telomere fusions and complete terminal deletions were the least frequent telomere aberrations and were not observed at significant levels in any of these experiments (Supplementary Figure 4 and Supplementary Table 7).

## DISCUSSION

Targeting telomerase and telomere maintenance mechanisms represents a promising therapeutic approach for various types of cancer [5, 6, 8]. However, assays comparing the efficiency and specificity of telomere-targeted compounds were lacking. Moreover, the known compounds that target telomerase or telomeres are limited in number. To address this point, we have recently developed a novel assay allowing comparison of different compounds for their ability to induce telomere dysfunction leading to chromosome loss [36].

It is well known that even transient telomere dysfunction can induce CIN in human cells [9]. Therefore, the activity of each compound can be evaluated based on its effect on CIN. To quantify this effect, we used two isogenic fibrosarcoma HT1080 cell lines with linear and circular HACs, each carrying an EGFP color marker. Specific destabilization of the linear HAC (containing telomeres) in response to drug treatment is consistent with specific targeting of telomeres. Conversely, destabilization of both linear and circular HACs indicates that a compound has off-target targets outside telomeres.

In our previous work, we applied this dual-HAC assay to analyze G-quadruplex ligands. That study identified two metal complexes G4 compounds, Cu-ttpy and Pt-ttpy, that exhibited a significant capacity to target telomeres as assessed by their potency at destabilizing the linear HAC [36]. It is worth noting that this family of metal-terpyridine complexes displays higher affinity and selectivity towards telomeric G4s *in vitro* [40, 46, 47]. A typical feature of these Pt(II)-terpyridine derivatives is their capacity to crosslink telomeric G4s via the formation of monoadducts with the purine bases in the G4-loops [39, 48]. Moreover, in cells, these compounds have been shown to target telomeres via platinum coordination (i.e., crosslinking) to telomeric DNA [34]. Thus, the cellular activity profiles of these platinum–terpyridine G4 ligands are expected to result from a combination of G4 targeting and Pt(II) crosslinking at purine bases (G, A).

In this study, we used the dual-HAC assay to analyze a series of six compounds, including the parent compound platinum-terpyridine Pt-tpy and five of its derivatives differing by the size of the aromatic terpyridine core [Pt-cpym, Pt-vpym, Pt-ttpy, Pt(PA)-tpy, and Pt-BisQ]. Binding of these complexes to a panel of oligonucleotides (G4 of various topologies and duplex DNA) revealed a significant preference for telomeric G4 sequences [39]. Moreover, that *in vitro* study revealed that the size and structure of the terpyridine-like core modulate both the G4 binding capacity and the formation of Pt-purine crosslinks. For instance, Pt-BisQ and Pt(PA)-tpy show poor-to-no capacity for Pt-crosslinking due to steric hindrance of the Pt-BisQ moiety for the former and blockage of the Pt atom by the phenylacetylene (PA) moiety for the latter. Our analysis reveals that four of these compounds, Pt-cpym, Pt-vpym, Pt-ttpy, and Pt-tpy, induce specific destabilization of a linear HAC. Of note, the two derivatives mentioned above with hindered Pt-crosslinking do not display significant activity in the assay suggesting that Pt-crosslinking might contribute at least partially to the telomere-selective effect observed with the linear HAC.

In our previous study we showed that for Pt-ttpy the telomere-selective effect was caused by specific targeting of telomeres that resulted in CIN [36]. Given the similar structure of the compounds studied here, a similar mechanism was proposed for Pt-cpym, Pt-vpym, and Pt-tpy. Indeed, after treatment of cells with these compounds, an increased number of DSBs at or near telomeres was observed. DSBs associated with telomeric sequences were confirmed by co-localization of γH2AX foci with the telomeric protein TRF2. Such telomere damage was proposed to lead to the formation of chromosome bridges in anaphase/telophase that we earlier observed for Pt-ttpy and Cu-ttpy [36], and ultimately resulted in chromosome mis-segregation. It is worth noting that we cannot exclude additional mechanisms of action for these G4 ligands, including transcriptional inactivation of genes important for chromosome segregation or induction of chromosomal DSBs as previously was described for other G4 stabilizers [49, 50].

Importantly, concentrations corresponding to LC_50_ of all four active compounds are in the μM range suggesting that they could potentially overcome the cisplatin resistance common in cancer cells [39]. Moreover, these terpyridine-derived platinum G4 ligands exhibit promising radiosensitization properties in human glioblastoma and lung cancer cells at subtoxic doses [51].

In conclusion, using our dual-HAC assay we identified three terpyridine platinum compounds, Pt-tpy, Pt-vpym and Pt-cpym, that induce a high level of chromosome instability (CIN) as previously reported for the related compound Pt-ttpy. CIN observed after treatment of cells with these compounds correlates with the formation of double-stranded DNA breaks predominantly localized proximal to telomeres. The telomere-associated DNA damage induced by these drugs leads to chromatin bridge formation in late mitosis and cytokinesis. This family of G4 ligands that induce telomere dysfunction and greatly increase chromosome mis-segregation rates are promising drug candidates for treatment of cancer alone or in combination with ionizing radiation.

## MATERIALS AND METHODS

### Cell lines and culture

The human fibrosarcoma (HT1080; ATCC^®^ CCL-121^™^) cell line (telomerase positive) [36] obtained from the American Type Culture Collection was authenticated both morphologically and by short tandem repeat analysis. The cell line was tested regularly to confirm lack of mycoplasma infection using a mycoplasma detection kit from InvivoGen. The HT1080 cells harboring either a circular alphoid^tetO^-HAC-EGFP [38] or a linear 21ΔqHAC-EGFP [37] were cultured in Dulbecco’s modified Eagle’s medium (DMEM) (Invitrogen) supplemented with 10% (v/v) fetal bovine serum (FBS, Clontech Laboratories, Inc.) at 37°C in a 5% CO_2_ atmosphere. For chromosome instability experiments, HT1080 cells were grown in medium containing 4 μg/ml Blasticidin S to prevent HAC loss prior to treatment with the drugs being tested (both linear and circular HACs contain the BS marker). After drug treatment, the cells were cultured in non-selective medium so that cells that have lost the HAC are able to grow. For mitotic abnormality experiments, the cells were not exposed to Blasticidin S because experiments were carried out in the parental HT1080 cell line with no HAC present.

### Compounds and treatments

Six different compounds [Pt-tpy and its five derivatives, Pt-cpym, Pt-vpym, Pt-ttpy, Pt(PA)-tpy, and Pt-BisQ], were used in our experiments. The experimental protocol was as previously described [36] (see details in Supplementary Materials). The compound concentration applied for measuring CIN was adjusted to the LC_50_ level for each compound. Concentrations of the compounds and lengths of treatments are presented in Supplementary Table 1. For each compound, the experiments on measuring EGFP-HAC loss were carried out in triplicate. The results were reproducible, and the standard deviations were small (for example, Pt-ttpy: SD ± 0.2%).

To study mitotic abnormalities induced by Pt-tpy and its derivatives in the HT1080 cell line, we used much lower concentrations of the drugs as at the LC_50_ only very small numbers of mitotic cells were seen. For these experiments, the cells were treated by noncytotoxic drug concentrations at dose based on published data [39] and confirmed experimentally for our cell lines (see Supplementary Table 1).

### FISH analysis of EGFP-HACs

The presence of the circular and linear HACs in an autonomous form was confirmed by FISH analysis as described previously [41, 52]. Metaphase cells were obtained by adding colcemid (Gibco) [36, 41].

### Telomere fluorescent *in situ* hybridization (Telo-FISH)

Metaphase spreads of drug-treated cells were prepared as described above and hybridized with a TelC-PNA telomere probe (Cy3-OO-TAACCCTAACCCTAACCC; PNA BIO). Slides were mounted with Vectashield^®^ Vibrance^™^ mounting media with DAPI and observed using a DeltaVision Elite imaging system. Z stacks were taken at 150 nm intervals and deconvolved. Images were analyzed with ImageJ software. Telomere aberrations scored were the following: telomere doublets, sister telomere losses, sister telomere fusions, terminal deletions. The numbers of metaphases analyzed in Telo-FISH experiments are reported in Supplementary Table 8.

### Flow cytometry

Analysis of EGFP intensities encoded by the HACs was performed by flow cytometry on a FACSCalibur instrument (BD Biosciences) using CellQuest acquisition software and analyzed statistically with FlowJo software. The cells were harvested by trypsin-treatment. A minimum of 4 × 10^4^ cells was analyzed for each cell sample.

### Calculation of the rate of spontaneous HAC loss and after compound treatment

The rate of HAC loss after cell treatment by a single dose of drug was calculated as previously described [41] (see details in Supplementary Materials).

### Cell viability test for measuring HAC loss in response to drug treatment

The LC_50_ for each compound was obtained as previously described [36] (Supplementary Table 1) (see details in Supplementary Materials). Experiments were carried out in triplicate for each drug.

### Cytokinesis-block micronucleus assay

Cytokinesis-block micronucleus formation assays were performed as described [53] with minor changes. After 72 hrs of cultivation, Cytochalasin B was added to a final concentration of 4.5 μg/ml for 24 hrs. The cells were trypsinized and 5 × 10^3^ cells were centrifuged onto cytoslides (Shandon, # 5991056) at 1,000 rpm for 1 min in a Cytospin 3 (Shandon). The slides were air-dried for 5 min, fixed with Diff-Quick fixative for 5 min, stained in Diff-Quick solution C (Eosin Y) (Electron Microscopy Sciences, # 26096) for 10 seconds, rinsed in distilled water and dried for 5 min. Coverslips were mounted with Vectashield^®^ Vibrance^™^ mounting media containing DAPI. Three slides from three independent treatments were prepared for each drug and for control vehicle treatment. The number of binucleated cells on each slide was scored along with the presence of micronuclei (MNi) or chromatin bridges (CBs) (Supplementary Table 8).

### Immunofluorescence

The number of γH2AX foci and the percentage of γH2AX foci associated with the telomeric sequences (identified by TRF2 staining) were calculated as previously described [36] (see details in Supplementary Materials). For each compound, 30 nuclei were analyzed (Supplementary Table 8).

For Figure 4 after drug treatment, the cells were fixed, permeabilized, and blocked as described above. The cells were stained with primary mouse monoclonal anti-LAP2 antibody (BD Biosciences, catalog no. #611000, dilution 1:200). Fluorophore-conjugated secondary antibodies were goat-anti-mouse Alexa 488, dilution 1:500 (Cell Signaling, catalog no. #4408S). The samples were mounted with Vectashield^®^ Vibrance^™^ mounting media with DAPI. Microscope images were acquired on a DeltaVision Core system (Applied Precision). Z-series were collected with a spacing of 0.25 μm, and image stacks were subsequently deconvolved using SoftWorx. Cells in telophase/cytokinesis were examined for the presence of chromatin bridges stained with DAPI and/or with LAP2.

### Statistical analysis

The statistical significance of comparisons between multiple groups was determined by unpaired two-tailed Student’s *t*-test with Bonferroni correction. *P* values of less than 0.05 were considered statistically significant. The data are presented in diagrams as mean, error bars correspond to 95% confidence interval. In all experiments, bars compare results of untreated cells (DMSO) with drug-treated cells.

## SUPPLEMENTARY MATERIALS



## Abbreviations

CIN: Chromosome instability
MNi: micronuclei
CBs: chromatid bridges
HAC: human artificial chromosome
TRF2: telomeric repeat binding factor 2
DSBs: double strand breaks
TD: telomere doublets
STL: single telomere loss
DMSO: dimetilsulfoxide
DNA: deoxyribonucleic acid
DMEM: Dulbecco’s modified Eagle’s medium
FBS: fetal bovine serum
