# Supplementary material for: Terpyridine platinum compounds induce telomere dysfunction and chromosome instability in cancer cells
Source: Oncotarget. 2021 Jul 20;12(15):1444–56. doi: 10.18632/oncotarget.28020 (PMC8310675; doi:10.18632/oncotarget.28020)
Supplement: Supplementary file 1 [file oncotarget-12-1444-s001.pdf]

Terpyridine platinum compounds induce telomere dysfunction and chromosome instability in cancer cells

SUPPLEMENTARY MATERIALS

Calculation of the rate of spontaneous HAC loss and after compound treatment

To calculate the rate of HAC loss after cell treatment by a single dose of drug, we used the formula $P_n = P_0 \times (1–R_{Drug})^{n1} \times (1–R_{Normal})^{n2}$ where P_0 is the percentage of HAC-containing cells in the population cultured under selection before drug treatment, P_n is the percentage of HAC-containing cells after *d* days in culture after drug treatment in absence of selection, n1 is the number of cell doublings that occurs during drug treatment, n2 is the number of cell doublings that occurs during culturing without selection after the drug treatment [41].

Cell viability test for measuring HAC loss in response to drug treatment

For each compound, the LC_{50} was determined using a MTS tetrazolium cell viability assay according to the manufacturer's instructions (CellTiter 96 AQueous Assay Reagent; Promega). Briefly, the CellTiter 96 AQueous One Solution Reagent was added to each well and incubated at 37°C for 3 hrs. Cell proliferation was determined by measuring the absorbance at 490 nm using a microtiter plate reader (Molecular Devices, Sunnyvale, CA, USA). The LC_{50} for each compound was obtained as previously described from the viability curves using GraphPad Prism 5 (Supplementary Table 1). Experiments were carried out in triplicate for each drug.

Immunofluorescence

For Figure 5, drug-treated cells were grown on coverslips, then rinsed with PBS, and fixed in 4% paraformaldehyde in PBS for 15 min at room temperature. Cells were rinsed twice quickly with PBS followed by incubation for 15 min with a last PBS wash at room temperature. Two-hundred milliliters of 5% BSA in PBS-TT (PBS containing 0.5% Tween-20, 0.1% Triton X-100) were added to the washed cells and incubated for 30 min in a humid chamber. Cells were rinsed once in PBS-T (PBS, containing 0.1% Tween-20) for 5 min. Two-hundred microliters of mouse *γH2AX antibody (*Abcam, catalog no. # 05-636, dilution 1:500) and rabbit TRF2 antibody (Santa Cruz Biotechnology, catalog no. # sc-9143n, dilution 1:200) in 1% BSA in PBS-TT were added for 2 hrs at room temperature in the humid chamber. The samples were washed three times for 5 min in PBS-T. Two-hundred microliters of secondary antibodies (goat-anti-mouse Alexa 488, dilution 1:500, goat-anti-rabbit Alexa 555, dilution 1:500, #4408S, # 4413S, Cell Signaling) were applied at room temperature in the humid chamber for 1 hr. The samples were washed three times in PBS-T for 5 min. The samples were counterstained with DAPI and mounted with Vectashield® Vibrance™ mounting media containing DAPI. Samples were analyzed using the Zeiss LSM 780 Microscopic System at the CRC, LRBGE Fluorescence Imaging Facility (NIH, Bethesda, MD). For each compound, 30 nuclei were analyzed (Supplementary Table 9). The number of γH2AX foci and the percentage of γH2AX foci associated with the telomeric sequences (identified by TRF2 staining) were calculated.

Compounds and treatments

Six different compounds [Pt-tpy and its five derivatives, Pt-cpym, Pt-vpym, Pt-ttpy, Pt(PA)-tpy, and Pt-BisQ], were used in our experiments. The experimental protocol was as follows. HT1080 cells containing either a circular or linear EGFP-HAC were maintained grown in the presence of Blasticidin S to select for the presence of the HAC. Approximately 1×10^5 cells were cultured either in the presence or absence of Blasticidin S selection in parallel with a third culture that was exposed to the agent under examination to test its effect on EGFP-HAC segregation. The compound concentration applied for measuring CIN was adjusted to the LC_{50} level for each compound (determined using a proliferation assay described below). Concentrations of the compounds and lengths of treatments are presented in Supplementary Table 1. After treatment, the compound was removed by performing three consecutive medium washes and the cells were subsequently grown without Blasticidin S selection for 14 days. At the end of the experiment, cells were collected and analyzed by flow cytometry to detect the proportion of cells that retained EGFP fluorescence. This served as a measure of EGFP-HAC stability following compound treatment. For each compound, the experiments on measuring EGFP-HAC loss were carried out in triplicate. The results were reproducible, and the standard deviations were small (for example, Pt-ttpy: SD ± 0.2%).

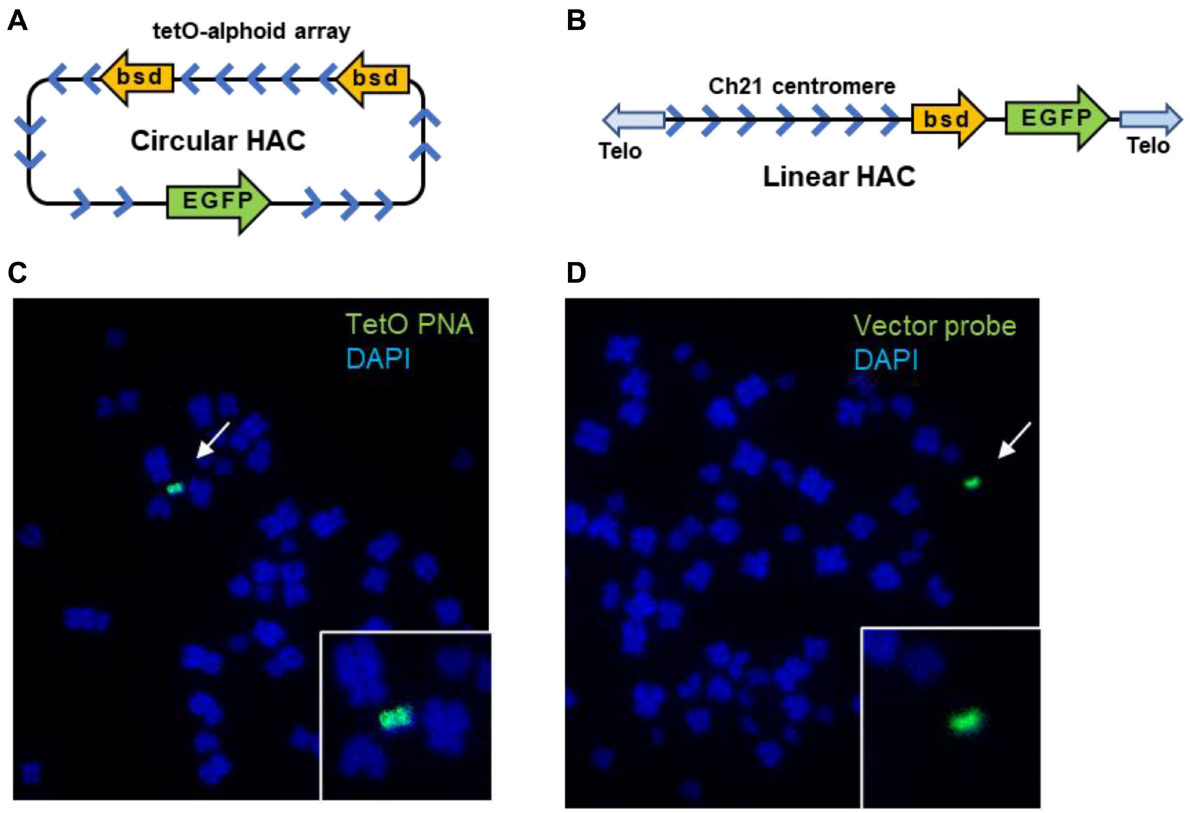

Supplementary Figure 1: Schemes of a circular alphoid^{tetO}-HAC (**A**) and a linear 21ΔqHAC (**B**). EGFP-a transgene. When it is expressed the cells are green. Telo-telomeric sequences in the linear HAC. bsd-a mammalian selectable marker blasticidin. alphoid^{tetO}-HAC contains approximately a 1.1 Mb tetO-alphoid array. A linear HAC contains approximately a 5 Mb alphoid array from the chromosome 21. (**C** and **D**) FISH analysis of the HACs in HT1080 cells. The HACs were visualized using a TetO probe for a circular HAC and a PNA probe for the vector part of the linear HAC as previously described [36]. Chromosomal DNA was counterstained with DAPI. The HACs are indicated by arrowhead.

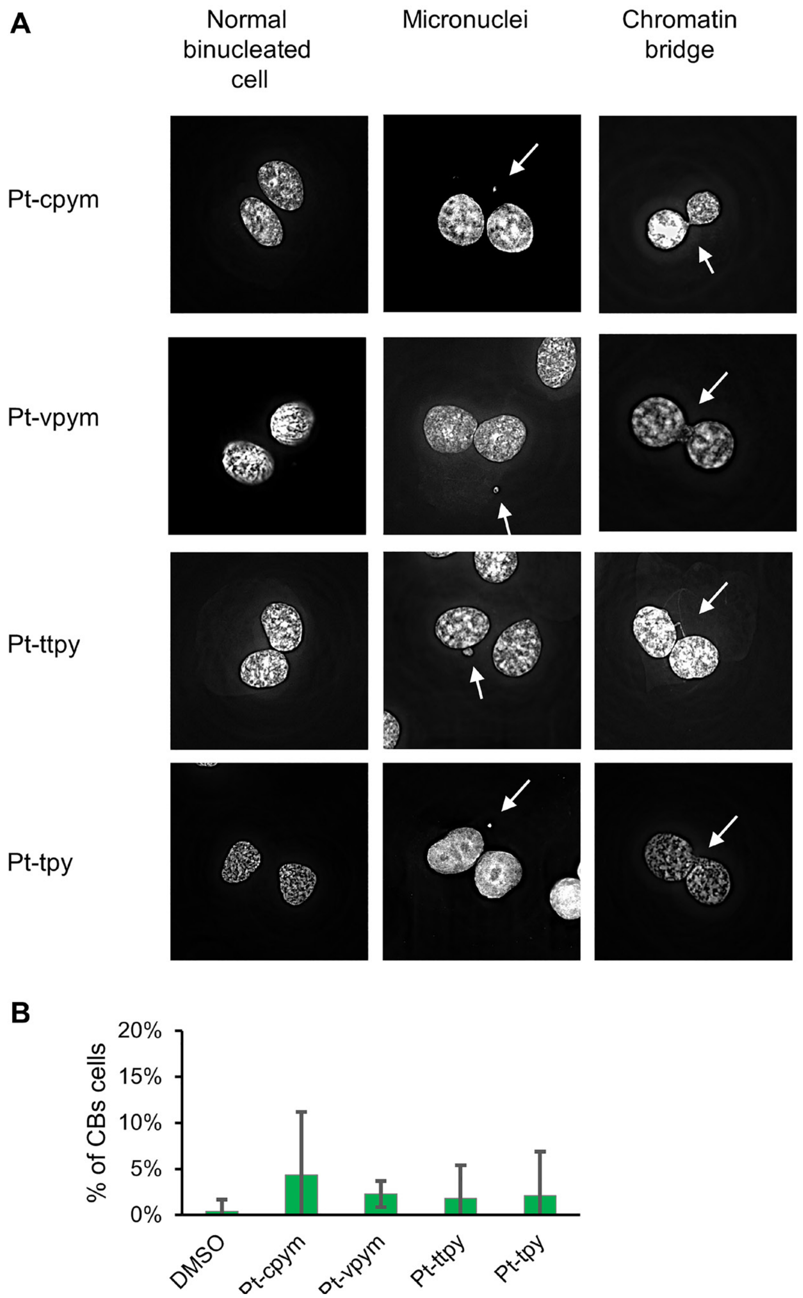

Supplementary Figure 2: (**A**) Examples of micronuclei (MNi) and chromatin bridges (CBs) formation in HT1080 cells after treatment by Pt-cpym, Pt-vpym, Pt-ttpy, and Pt-tpy. (**B**) The percentage of CBs. Error bars correspond to the 95% confidence intervals (95% CI) of four replicates.

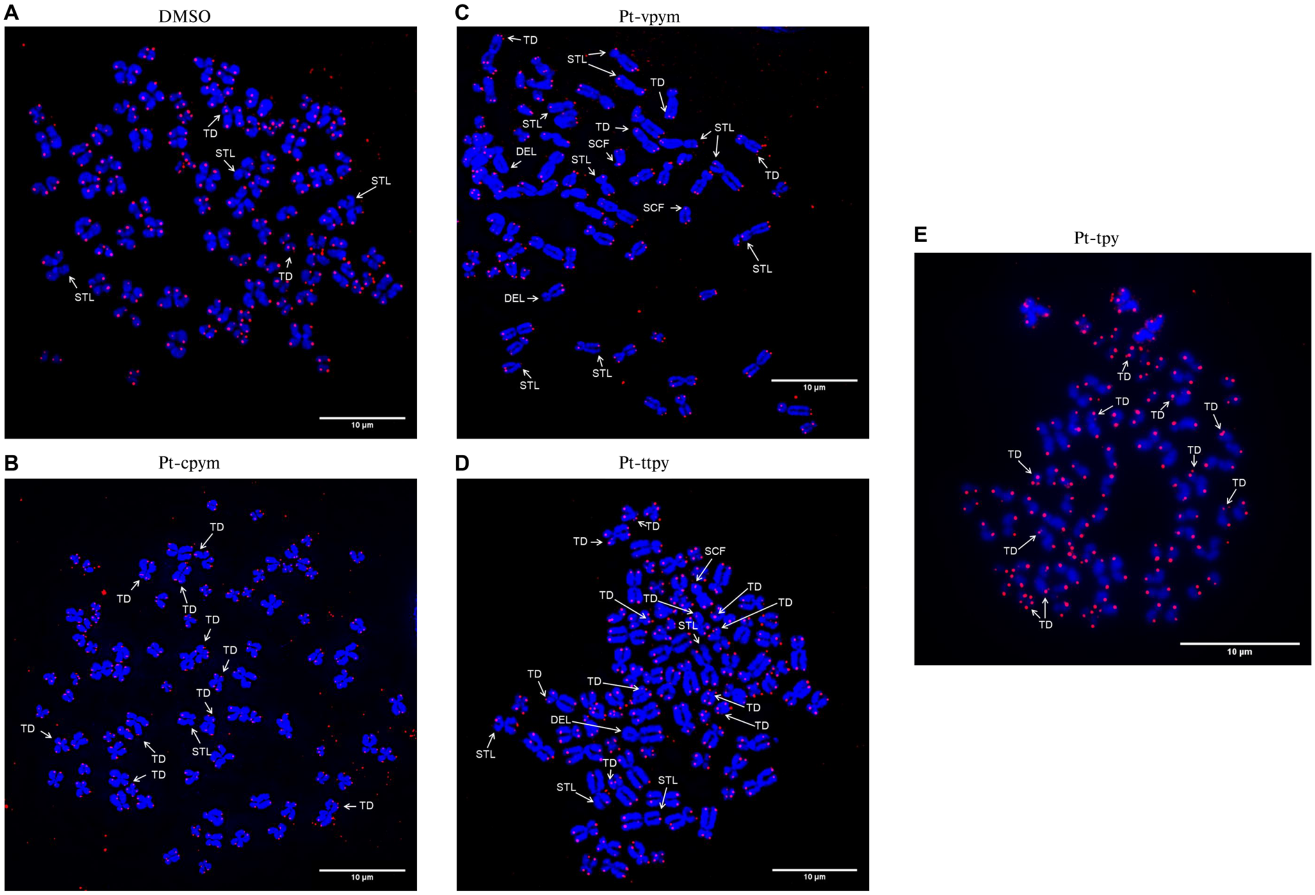

Supplementary Figure 3: Metaphase spreads of HT1080 cells with different telomere aberrations after treatment by DMSO (**A**), Pt-cpym (**B**), Pt-vpym (**C**), Pt-ttpy (**D**) and Pt-tpy (**E**) compounds. Metaphase spreads were hybridized with a telomeric PNA probe (in red) and then counterstained with DAPI (in blue). TD-telomere doublets; STL-single telomere loss; SCF-sister chromatid fusion; DEL-telomere deletion.

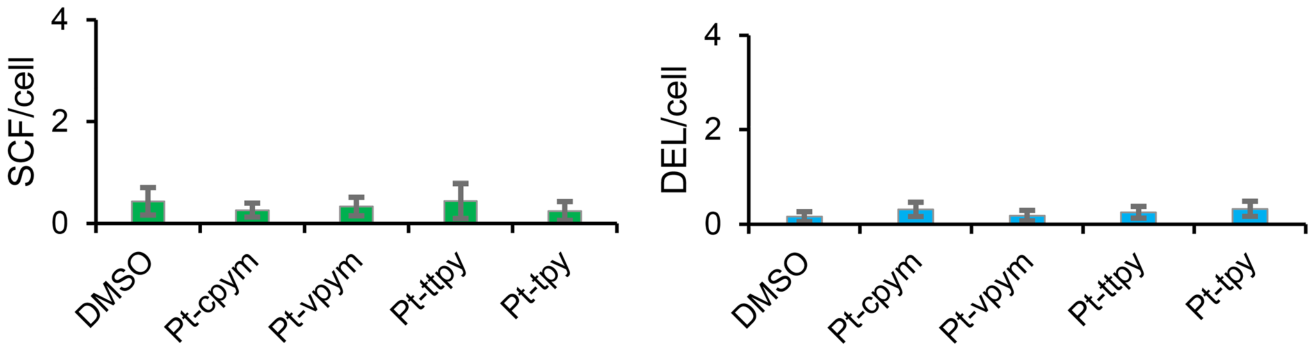

Supplementary Figure 4: Histograms show the percentages of chromosomes with the indicated telomere damage per cell (SCFs and DELs) detected in metaphase spreads of treated versus untreated cells.

Supplementary Table 1: Concentrations of the drugs used in this study

Drug	HAC loss experiments LC50	Natural chromosomes damage experiments
Pt-cpym	10 µM	2 µM
Pt-vpym	2 µM	0.4 µM
Pt-ttpy	10 µM	2 µM
Pt-tpy	100 µM	20 µM
Pt(PA)-tpy	0.5 µM	
Pt-BisQ	100 µM	

Supplementary Table 2: Effect of drug treatment on a linear and a circular HAC mis-segregation rate

Drug	Linear HAC loss rate per cell division	Circular HAC loss rate per cell division	*p* value
Control	1.0%	0.9%	1
Pt-cpym	38.8%	9.7%	**0.0099**
Pt-vpym	35.6%	3.0%	**0.0008**
Pt-ttpy	37.9%	4.9%	**0.0043**
Pt-tpy	33.5%	5.3%	**0.0032**
Pt(PA)-tpy	7.6%	4.1%	0.305
Pt-BisQ	4.1%	2.3%	1

A data set was obtained by the average of three independent experiments for each drug.

Supplementary Table 3: The rates of mitotic phenotypes in the Cytokinesis-block micronucleus assay after drug treatment in comparison with vehicle (DMSO) treatment

	Drug	Percentage of the phenotype	95% CI	Fold change	*p* value
**Normal cells**	DMSO	87%	81–93%		
	Pt-cpym	59%	34–85%	0.69	**0.044**
	Pt-vpym	69%	59–79%	0.80	**0.012**
	Pt-ttpy	63%	58–68%	0.73	**0.0008**
	Pt-tpy	61%	56–67%	0.71	**0.0007**
**Micronuclei**	DMSO	13%	8–17%		
	Pt-cpym	36%	18–55%	2.8	**0.025**
	Pt-vpym	28%	19–38%	2.2	**0.013**
	Pt-ttpy	35%	26–44%	2.8	**0.0025**
	Pt-tpy	37%	34–39%	2.9	**0.0002**
**Chromatin bridges**	DMSO	0.4%	0–2%		
	Pt-cpym	4%	0–11%	7.5	0.32
	Pt-vpym	2%	1–4%	3.9	0.073
	Pt-ttpy	2%	0–5%	3.1	0.97
	Pt-tpy	2%	0–7%	3.7	0.98

Average percentages, 95% confidence intervals (95% CI), fold change in comparison to control and *p* values of *t*-test with Bonferroni correction presented. A data set was obtained by the average of four independent experiments for each drug.

Supplementary Table 4: The rates of chromatin bridges formation in late mitosis after drug treatment in comparison with vehicle (DMSO) treatment

Drug	Percentage of the phenotype	95% CI	Fold change	*p* value
DMSO	20%	16–24%		
Pt-cpym	40%	28–52%	2.1	**0.033**
Pt-vpym	37%	25–49%	1.9	**0.024**
Pt-ttpy	38%	26–50%	1.9	**0.048**
Pt-tpy	35%	25–45%	1.8	**0.047**

Average percentages, 95% confidence intervals (95% CI), fold change in comparison to control and *p* values of *t*-test with Bonferroni correction presented. A data set was obtained by the average of three independent experiments for each drug.

Supplementary Table 5: The quantity of γH2AX foci after drug treatment in comparison with vehicle (DMSO) treatment

Drug	γH2AX foci number per cell	95% CI	Fold increase	*p* value
DMSO	5.3	2.8–7.9		
Pt-cpym	51.1	36.3–65.9	9.6	**<0.001**
Pt-vpym	61.5	49–74	11.6	**<0.001**
Pt-ttpy	54.5	39.3–69.7	10.3	**<0.001**
Pt-tpy	59.9	48.5–71.2	11.3	**<0.001**

Average quantities, 95% confidence intervals (95% CI), fold change in comparison to control and *p* values of *t*-test with Bonferroni correction presented. A data set was obtained by the average of three independent experiments for each drug.

Supplementary Table 6: The percentage of telomeres colocalized with γH2AX DNA damage signal after drug treatment in comparison with vehicle (DMSO) treatment

Drug	Percentage of damaged TFR2 foci	95% CI	Fold increase	*p* value
DMSO	1.0%	0.2–1.8%		
Pt-cpym	11.5%	8.1–15%	11.5	**<0.001**
Pt-vpym	8.6%	6.6–10.7%	8.6	**<0.001**
Pt-ttpy	13.4%	9.9–16.8%	13.4	**<0.001**
Pt-tpy	13.8%	11–16.5%	13.8	**<0.001**

Average percentages, 95% confidence intervals (95% CI), fold change in comparison to control and *p* values of *t*-test with Bonferroni correction presented. A data set was obtained by the average of three independent experiments for each drug.

Supplementary Table 7: The augmentation of telomere aberrations on metaphase spreads after drug treatment in comparison with vehicle (DMSO) treatment

	Drug	Number of aberrations per cell	95% CI	Fold increase	*p* value
**Total damaged chromosomes**	DMSO	4.1	3.8 - 4.5		
	Pt-cpym	8.8	7.9 - 9.7	2.1	**<0.0001**
	Pt-vpym	9.9	9.2 - 10.7	2.4	**<0.0001**
	Pt-ttpy	10.2	9.4 - 11.1	2.5	**<0.0001**
	Pt-tpy	9.6	8.8 - 10.4	2.3	**<0.0001**
**Telomere doublets**	DMSO	2.1	1.7 - 2.5		
	Pt-cpym	4.8	3.9 - 5.7	2.3	**<0.0001**
	Pt-vpym	6	5.4 - 6.7	2.9	**<0.0001**
	Pt-ttpy	6	5.2 - 6.8	2.9	**<0.0001**
	Pt-tpy	5.6	4.9 - 6.3	2.7	**<0.0001**
**Single telomere loss**	DMSO	1.5	1.2 - 1.7		
	Pt-cpym	3.4	2.8 - 4	2.3	**<0.0001**
	Pt-vpym	3.4	2.8 - 4	2.3	**<0.0001**
	Pt-ttpy	3.5	3 - 4.1	2.4	**<0.0001**
	Pt-tpy	3.4	2.7 - 4.1	2.3	**<0.0001**
**Sister Chromatid Telomeric Fusions**	DMSO	0.4	0.2-0.7		
	Pt-cpym	0.3	0.1 - 0.4	0.6	1
	Pt-vpym	0.3	0.2 - 0.5	0.8	1
	Pt-ttpy	0.4	0.1 - 0.8	1	1
	Pt-tpy	0.2	0.1 - 0.4	0.6	1
**Telomere deletions**	DMSO	0.2	0.1 - 0.3		
	Pt-cpym	0.3	0.2 - 0.5	2	0.39
	Pt-vpym	0.2	0.1 - 0.3	1.1	1
	Pt-ttpy	0.3	0.1 - 0.4	1.6	1
	Pt-tpy	0.3	0.2 - 0.5	2	0.36

Average quantities, 95% confidence intervals (95% CI), fold change in comparison to control and *p* values of *t*-test with Bonferroni correction presented. A data set was obtained by the average of three independent experiments for each drug.

Supplementary Table 8: The numbers of analyzed nuclei or metaphase spreads

Drug	Number of cells evaluated in		
	**Micronucleation test**	**DNA damage assay**	**Telomere aberrations assay**
Control	334	30	60
Pt-cpym	339	30	60
Pt-vpym	357	30	60
Pt-ttpy	326	30	60
Pt-tpy	328	30	60
